# Supplementary material for: On the quality of commercial chemical vapour deposited hexagonal boron nitride
Source: Nat Commun. 2024 May 28;15:4518. doi: 10.1038/s41467-024-48485-w (PMC11133478; doi:10.1038/s41467-024-48485-w)
Supplement: Supplementary file 1 — Supplementary Information [file 41467_2024_48485_MOESM1_ESM.pdf]

SUPPLEMENTARY INFORMATION

On the quality of commercial chemical vapour deposited hexagonal boron nitride

Yue Yuan[1], Jonas Weber[1], Junzhu Li[1], Bo Tian[1], Yinchang Ma[1], Xixiang Zhang[1], Takashi Taniguchi[2], Kenji Watanabe[3], Mario Lanza[1,*]

[1] Materials Science and Engineering Program, Physical Science and Engineering Division, King Abdullah University of Science and Technology (KAUST), Thuwal 23955-6900, Saudi Arabia
[2] International Center for Materials Nanoarchitectonics, National Institute for Materials Science, 1-1 Namiki, Tsukuba 305-0044, Japan
[3] Research Center for Functional Materials, National Institute for Materials Science, 1-1 Namiki, Tsukuba 305-0044, Japan

[*] Corresponding author Email: mario.lanza@kaust.edu.sa

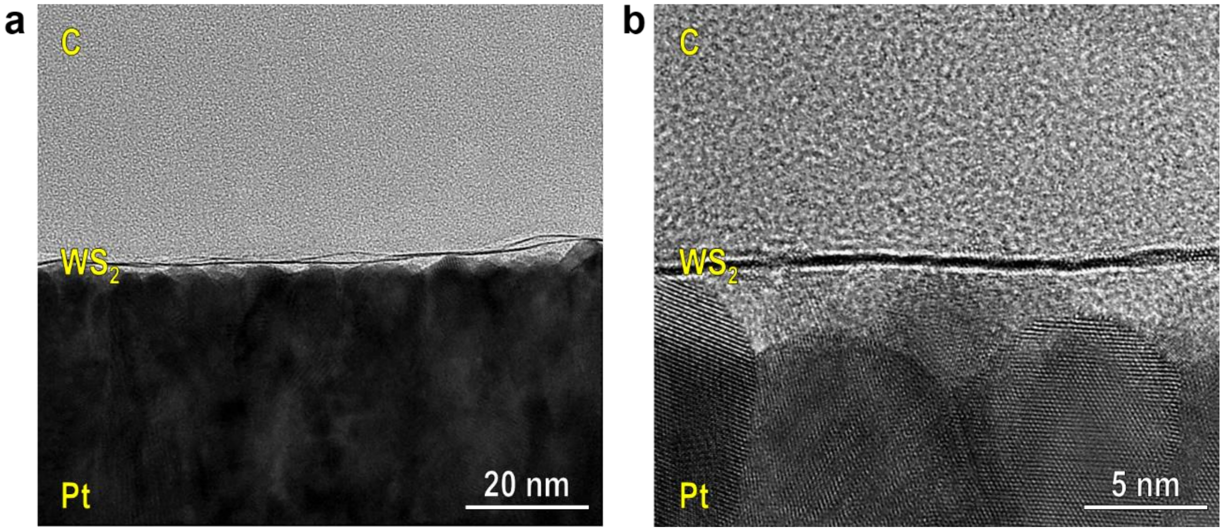

Supplementary Figure 1 | Cross-sectional TEM images of monolayer 2D material on evaporated metallic film. a, low magnification TEM image of CVD-grown monolayer WS_2 transferred on electron-beam evaporated Pt film. **b**, high magnification TEM image of one portion of the image in panel **a**. A clear difference in gaps between the WS_2 and Pt substrate can be observed.

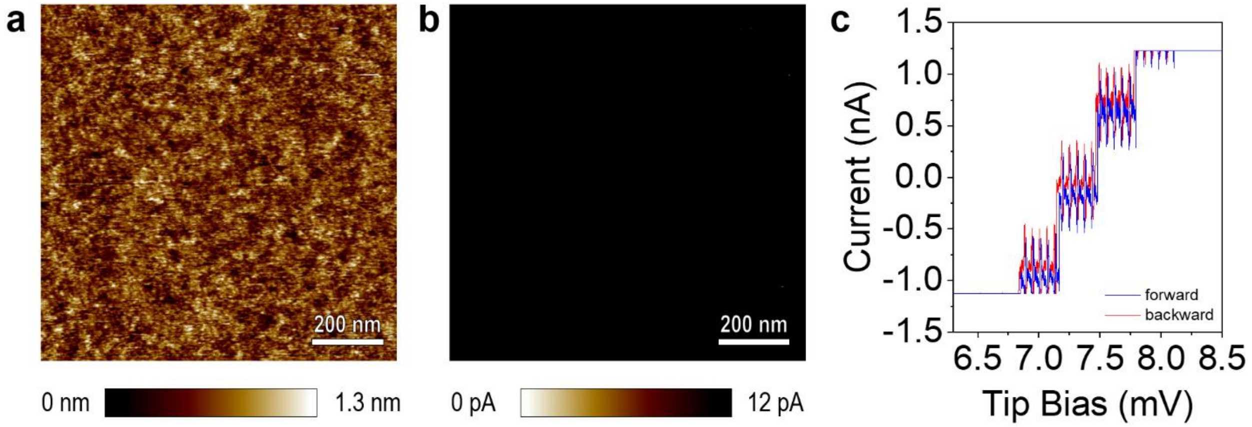

Supplementary Figure 2 | CAFM characterization of 5 nm Ru / 30 nm Ta / 300 nm SiO_2 / Si substrate. a, topography map of the substrate in the size of 1 μm × 1 μm, its RMS surface roughness is 0.310 nm. **b,** corresponding CAFM current map collected without applying bias, current is observed everywhere. **c,** forward and backward I-V curves with 20480 points in each curve, taken at random position on the substrate. The stepped nature of the I-V curve in the central part of panel **c** is related to the minimum step of the voltage source of the Bruker Dimension Icon CAFM, which is ~0.3 mV. For example, we can tell the voltage source to set the voltage to 1 mV, but we cannot tell the voltage source to set 1.215478953 mV. The voltage source has not such a high precision, it works in small steps. The noisy current signal within each step is related to small resistance fluctuations in the tip-sample system, due to by instabilities of the tip-sample contact force and electrical noise in the voltage applied by the CAFM. These two behaviours are characteristic when the CAFM tip is measuring on a metallic sample, and they have been also observed in other CAFMs, such as Multimode V [17].

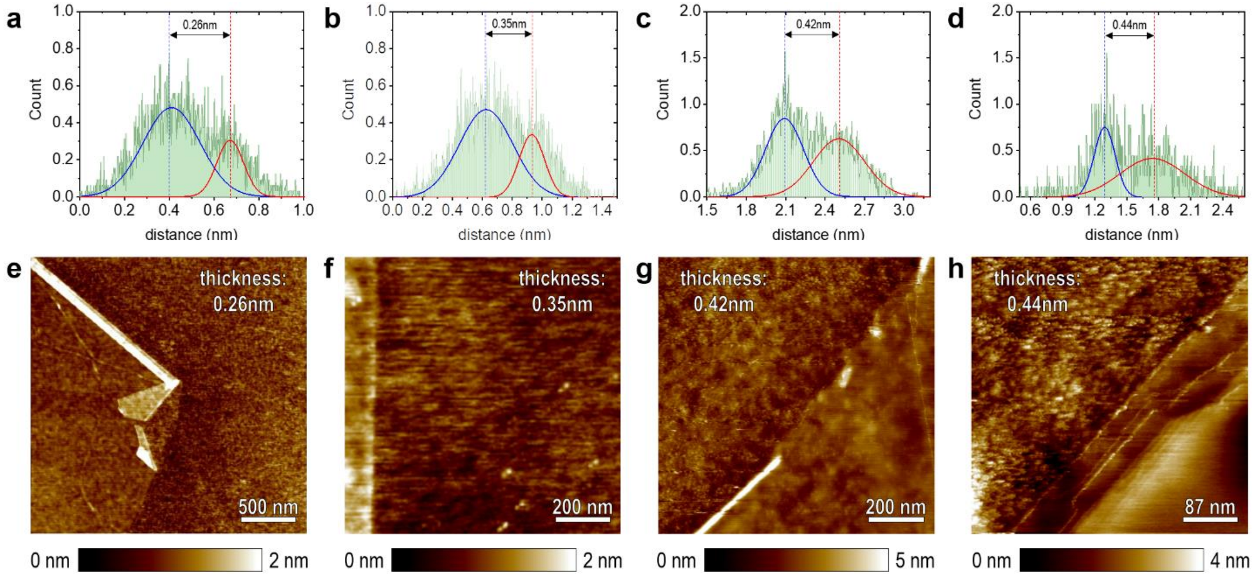

Supplementary Figure 3 | Topography and thickness analysis of mechanically exfoliated monolayer h-BN. The bottom row shows the original AFM topographic maps collected at the edge of four different mechanically exfoliated monolayer flakes. The top row shows the histogram distribution, all of them showing two peaks, one corresponding to the substrate and one corresponding to the surface of the monolayer h-BN. The thickness in all cases is indicated as the distance between the two peaks, and it is always around the values expected for monolayer h-BN (0.33 nm).

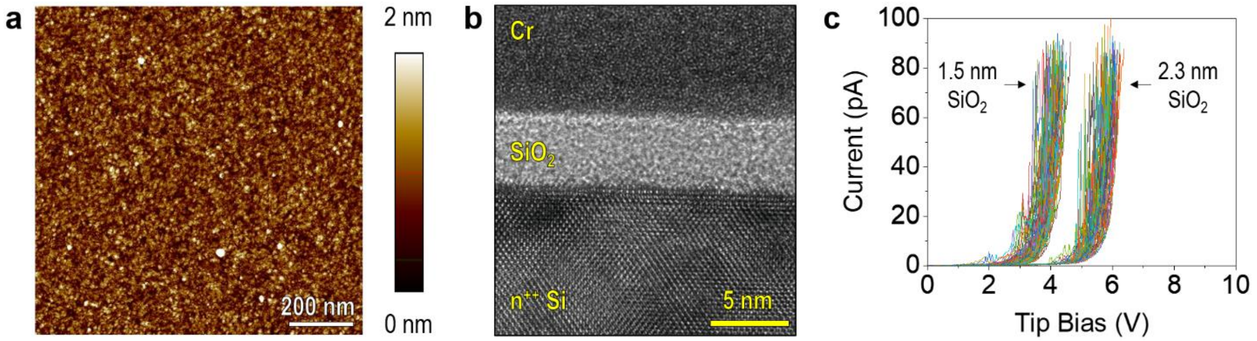

Supplementary Figure 4 | CAFM and TEM characterization of thermal SiO_2 samples. a, AFM topography map of a 1.5-nm-thick thermal SiO_2 / n^{++} Si sample. **b**, cross-sectional TEM image of the thermal SiO_2 sample. **c**, CAFM 100 forward I-V curves for 1.5-nm-thick SiO_2 / n^{++} Si (left) and 3.2-nm-thick SiO_2 /n^{++} Si (right), collected at different random positions (in a matrix of 10 µm × 10 µm), current is limited at 100 pA.

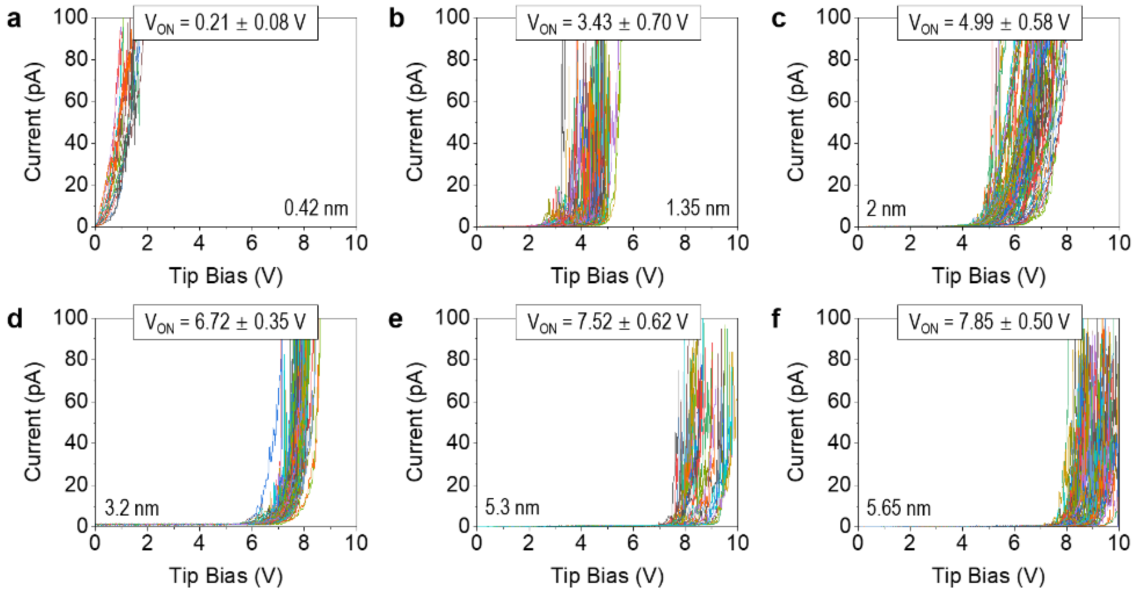

Supplementary Figure 5 | CAFM I-V curves collected on mechanically exfoliated h-BN flakes, with different thicknesses. a-f, Plots containing 100 I-V curves collected on mechanically exfoliated monolayer with thicknesses of 0.42, 1.35, 2, 3.2, 5.3 and 5.65 nm, at different random locations of their surfaces.

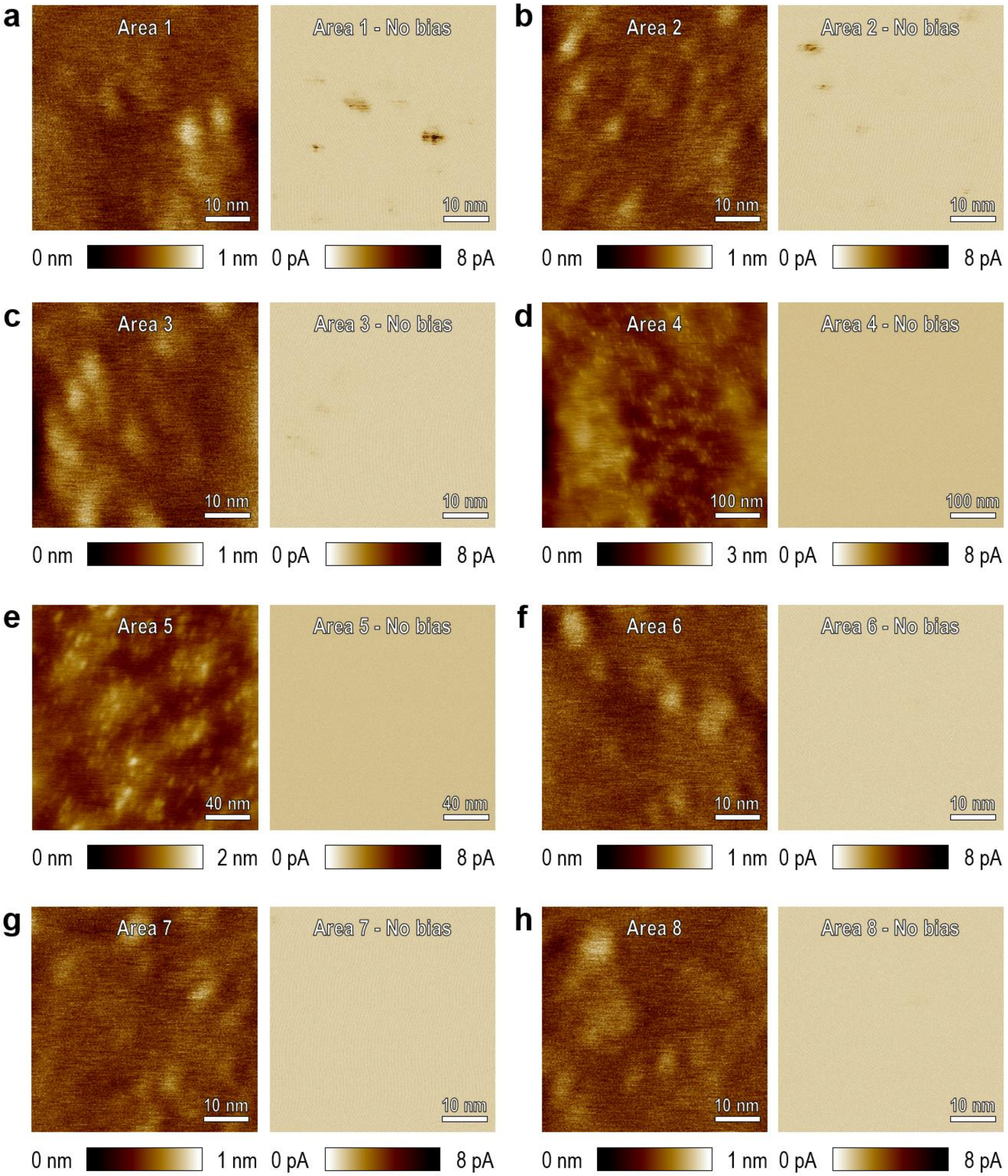

Supplementary Figure 6 | CAFM characterization of mechanically exfoliated monolayer h-BN on Ru substrate. High-resolution CAFM topography and current maps simultaneously collected without applying any bias at eight different areas. Areas 1-3 (panels **a**-**c**) are near the edge of the flake, and areas 4-8 (panels **d**-**h**) are far from the edge.

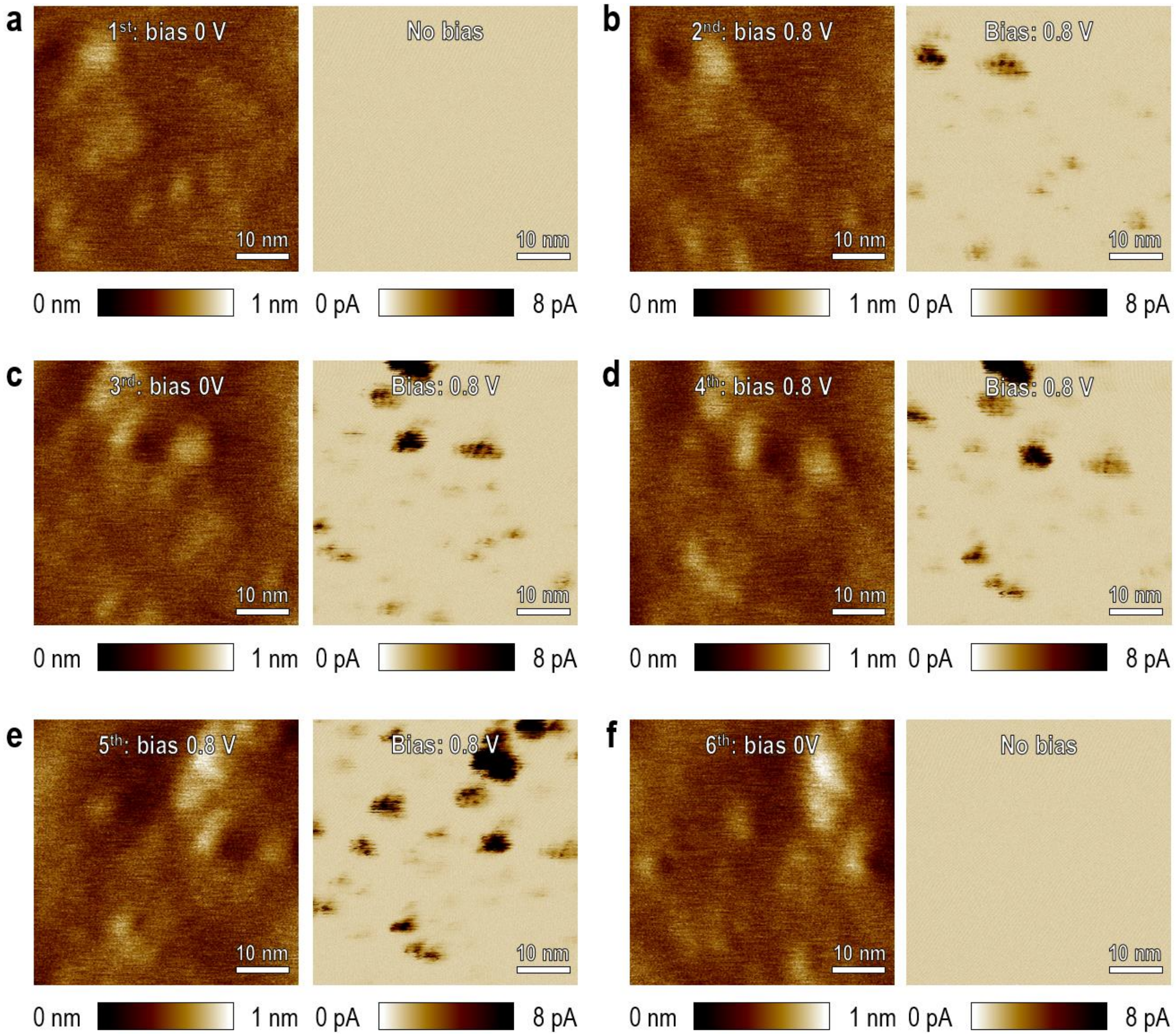

Supplementary Figure 7 | CAFM characterization of mechanically exfoliated monolayer h-BN on Ru substrate. Six groups of high-resolution consecutive CAFM topography and current maps collected at the same area, in a size of 50 nm × 50 nm. Panel **a,** the first CAFM scan, collected without bias. Panel **c-e,** four consecutive CAFM scans, collected with a tip bias 0.8 V. Panel **f,** the last CAFM scan, collected without bias.

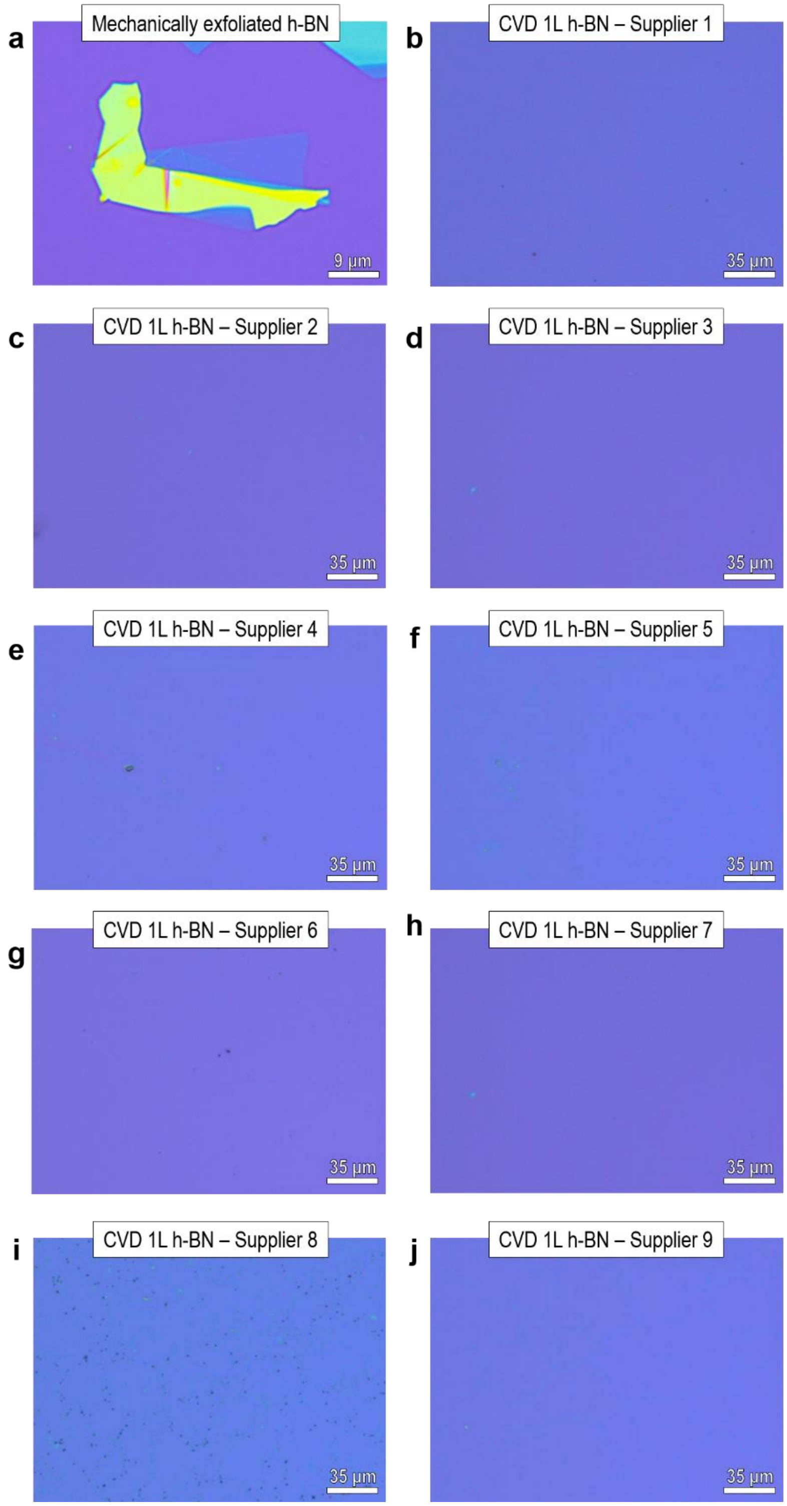

Supplementary Figure 8 | Optical microscope images of mechanically exfoliated h-BN and CVD-grown h-BN samples labelled as monolayer from Suppliers 1-9, transferred on 300 nm SiO_2 / Si substrates. a, optical microscope image of mechanically exfoliated h-BN, where large thickness fluctuation can be easily observed by the contrast. **b-j**, optical microscope images of CVD-grown h-BN samples labelled as monolayer from Suppliers 1-9.

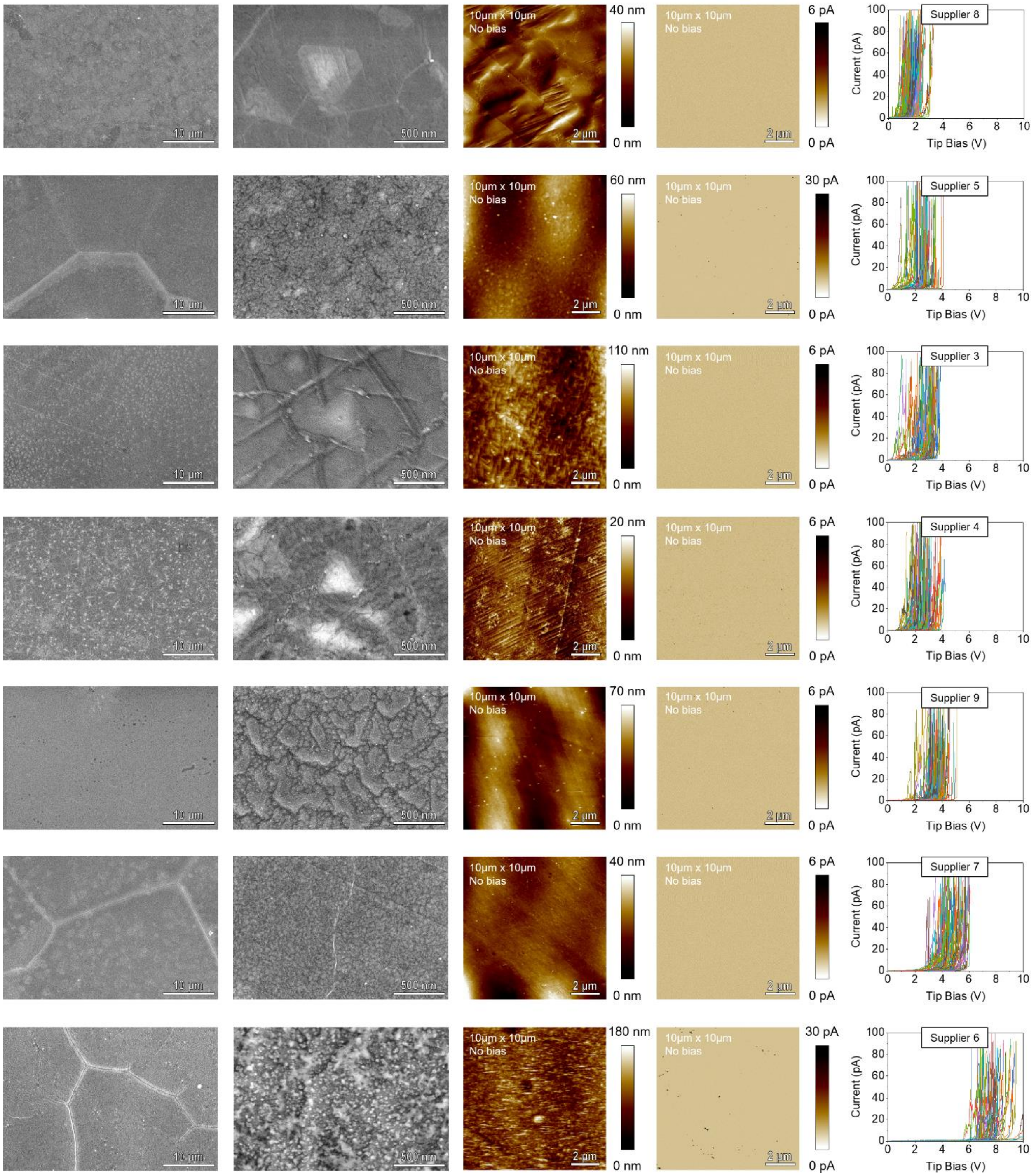

Supplementary Figure 9 | SEM and CAFM characterization of CVD-grown h-BN samples labelled as monolayer from Suppliers 3, 4, 5, 6, 7, 8 and 9. Left two columns show the SEM images of each sample, in different magnifications. The central two columns shows the CAFM topography maps and current maps at 0V, and the right column shows 100 forward I-V curves for each sample collected at different random positions (in a matrix of 10 μm × 10 μm).

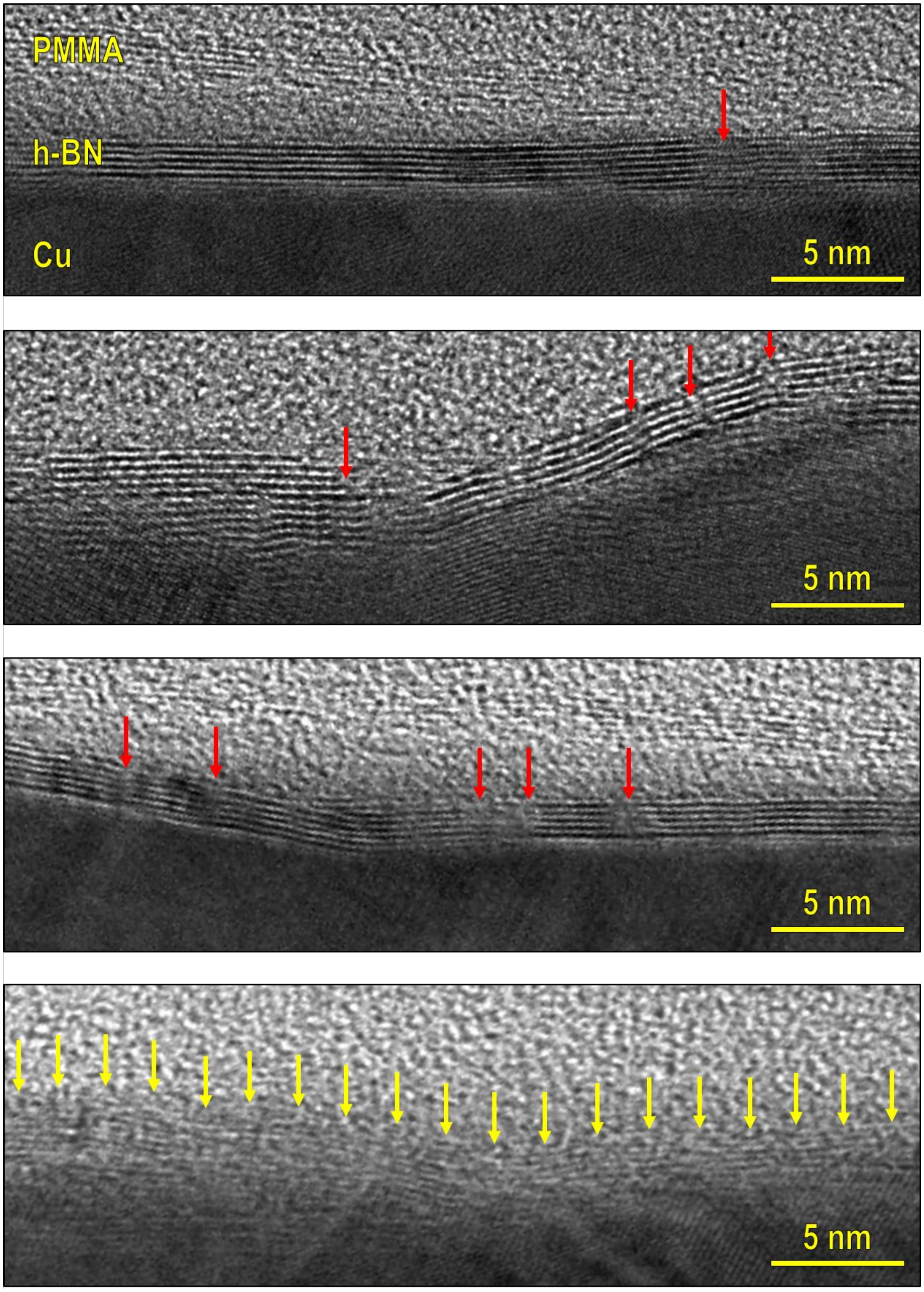

Supplementary Figure 10 | Cross-sectional TEM images of CVD-grown h-BN samples on Cu, labelled as monolayer, from Supplier 1. The PMMA served as protective coating during the FIB cut. The layered structure is obvious, with van der Waals gap between each layer.

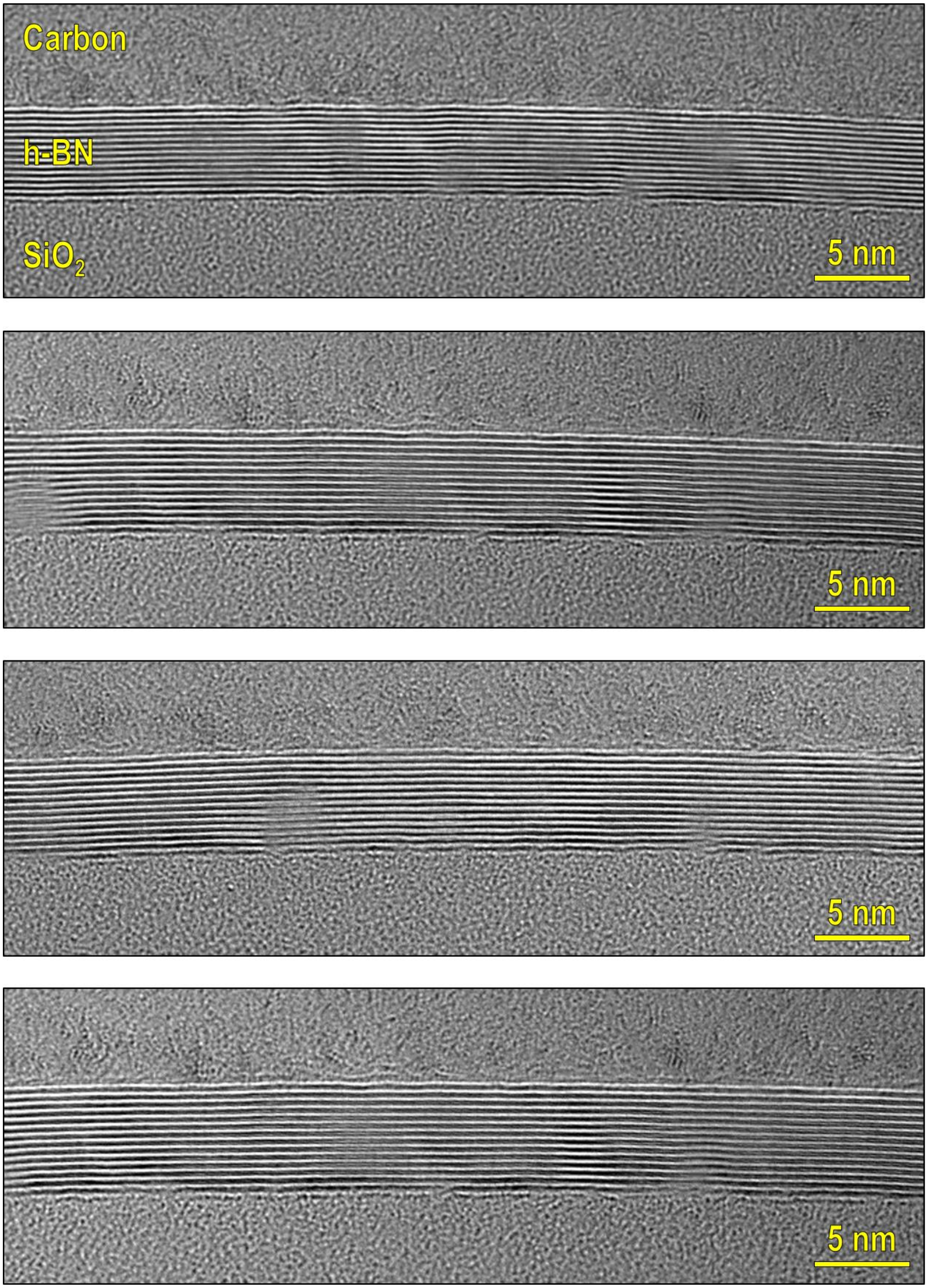

Supplementary Figure 11 | Cross-sectional TEM images of one mechanically exfoliated h-BN flake from NIMS. Continuous and clear layer structure with almost no defect between the h-BN layers can be observed.

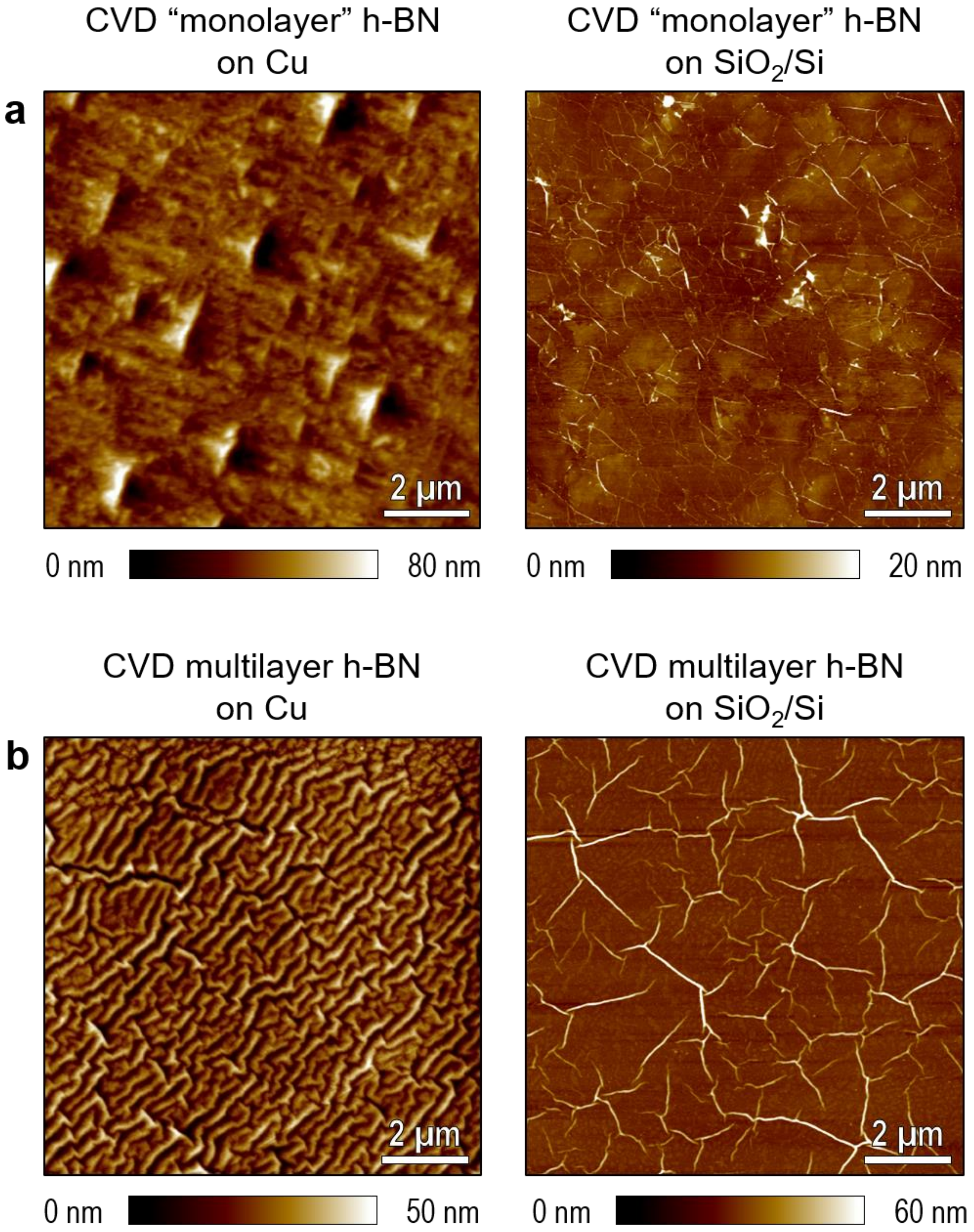

Supplementary Figure 12 | Surface roughness of commercial CVD-grown h-BN. a, Comparison of the surface roughness of a commercial CVD-grown h-BN on the Cu foil, labelled as monolayer from Supplier 1, on which it was grown and after being transferred on ultra-flat SiO_2/Si wafers. **b,** Comparison of the surface roughness of a commercial CVD-grown multilayer h-BN on the Cu foil on which it was grown and after being transferred on ultra-flat SiO_2/Si wafers.

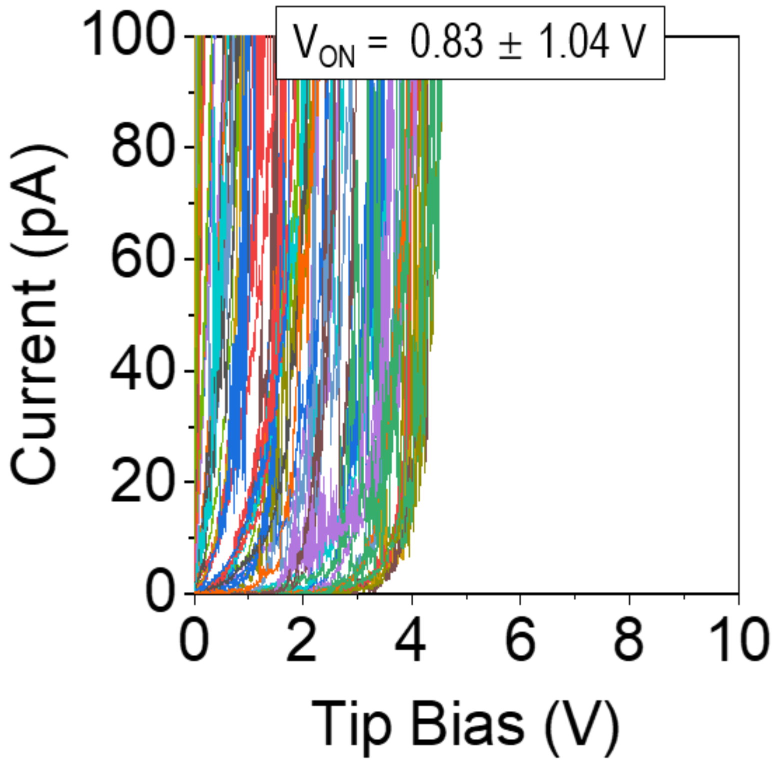

Supplementary Figure 13 | CAFM characterization of CVD-grown h-BN sample, labelled as monolayer, from Supplier 1, transferred on a 5 nm Ru / 30 nm Ta / 300 nm SiO_2 / Si substrate. 100 forward I-V curves collected with a 100 pA current limitation.

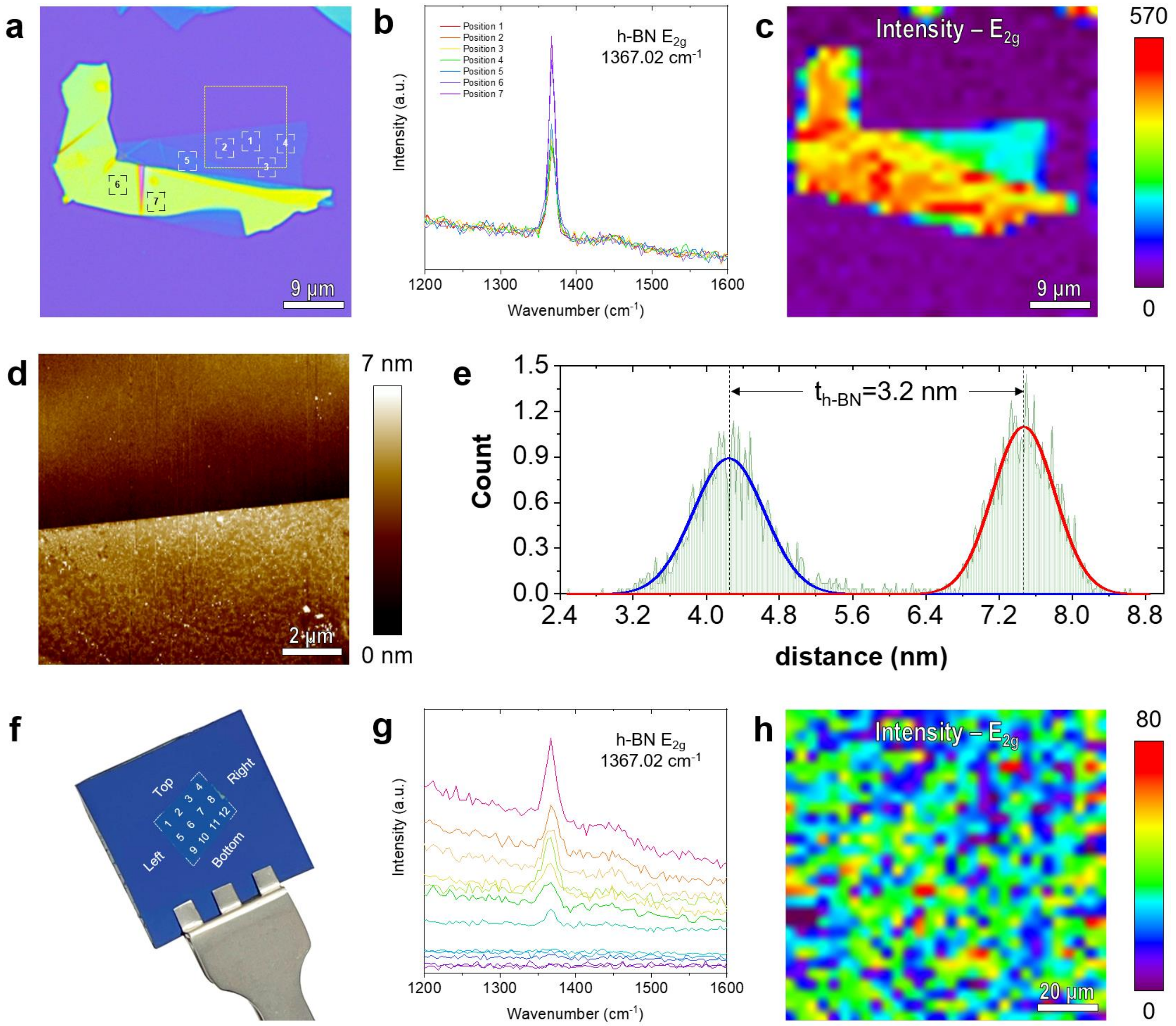

Supplementary Figure 14 | Raman characterization of mechanically exfoliated h-BN and CVD-grown h-BN sample labelled as monolayer from Supplier 1, transferred on 300 nm SiO_2 / Si substrates. a-e, Raman and CAFM maps of mechanically exfoliated h-BN flake. **a,** optical microscopy image of one mechanically exfoliated h-BN flake. **b,** Typical Raman spectrum at 7 positions marked in **a**. A sharp E_{2g} band of h-BN at 1367.02 cm^{-1} with a FWHM of 12 cm^{-1} was observed. **c,** a 45 µm × 45 µm Raman map of the intensity of h-BN E_{2g} band. **d,** AFM topography map collected at the edge of the target mechanically exfoliated h-BN flake, which is the area that marked with yellow dash line in **a**. **e,** histogram distribution in **d**, the distance between two peaks is 3.2 nm, indicating the thickness of this h-BN flake. **f-h,** Raman experiments of CVD-grown h-BN labelled as monolayer from Supplier 1. **f,** photography of 5 mm × 8 mm CVD-grown h-BN labelled as monolayer from Supplier 1 on a SiO_2 (300 nm) / Si substrate after transfer. **g,** typical Raman spectrum at 12 positions indicated in **f**. **h,** a 100 µm × 100 µm Raman map of the intensity of h-BN E_{2g} band.

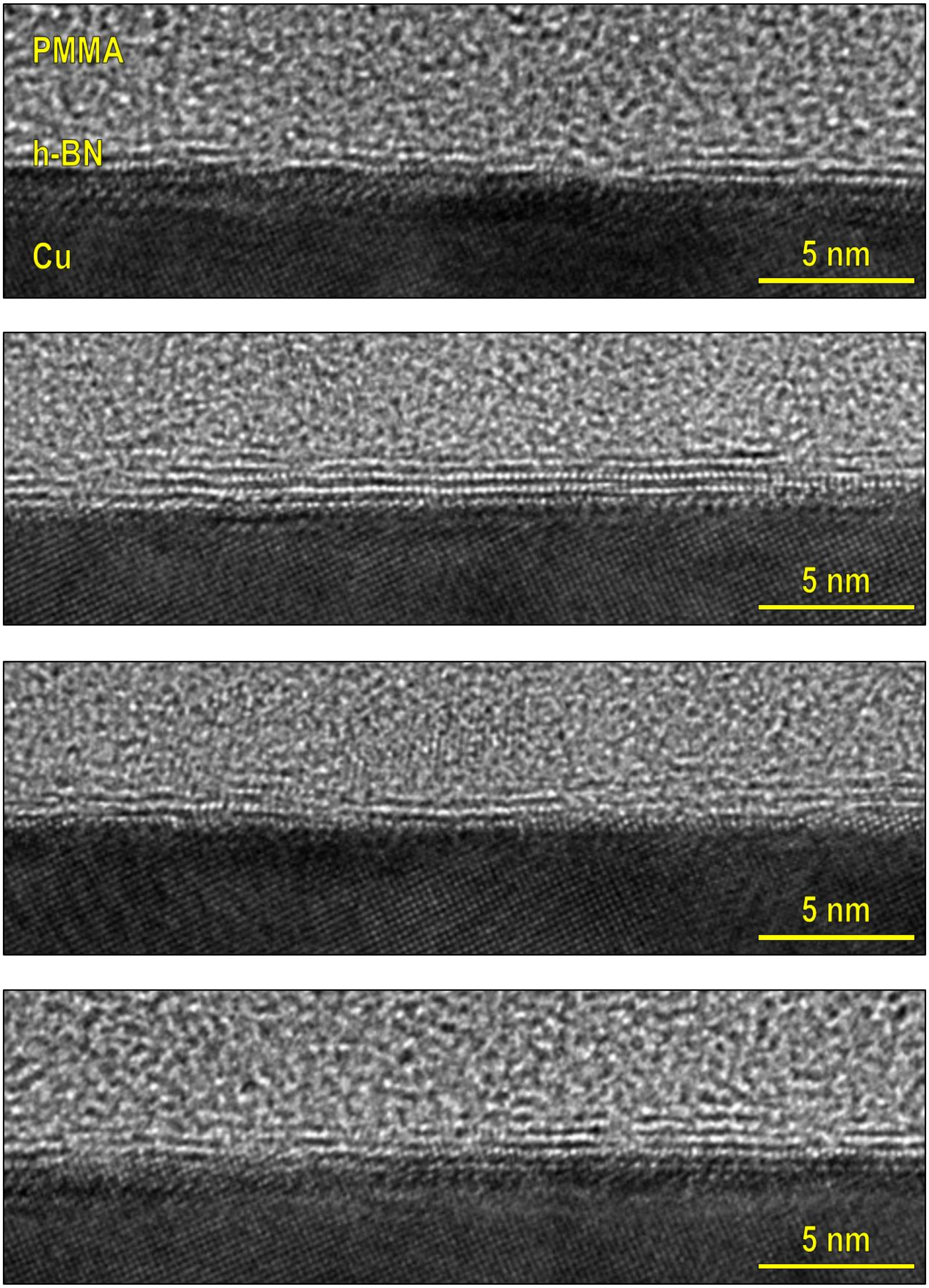

Supplementary Figure 15 | Cross-sectional TEM images of CVD-grown h-BN sample on Cu, labelled as monolayer from Supplier 2. The PMMA served as protective coating during the FIB cut. The layered structure is obvious, with van der Waals gap between each layer.

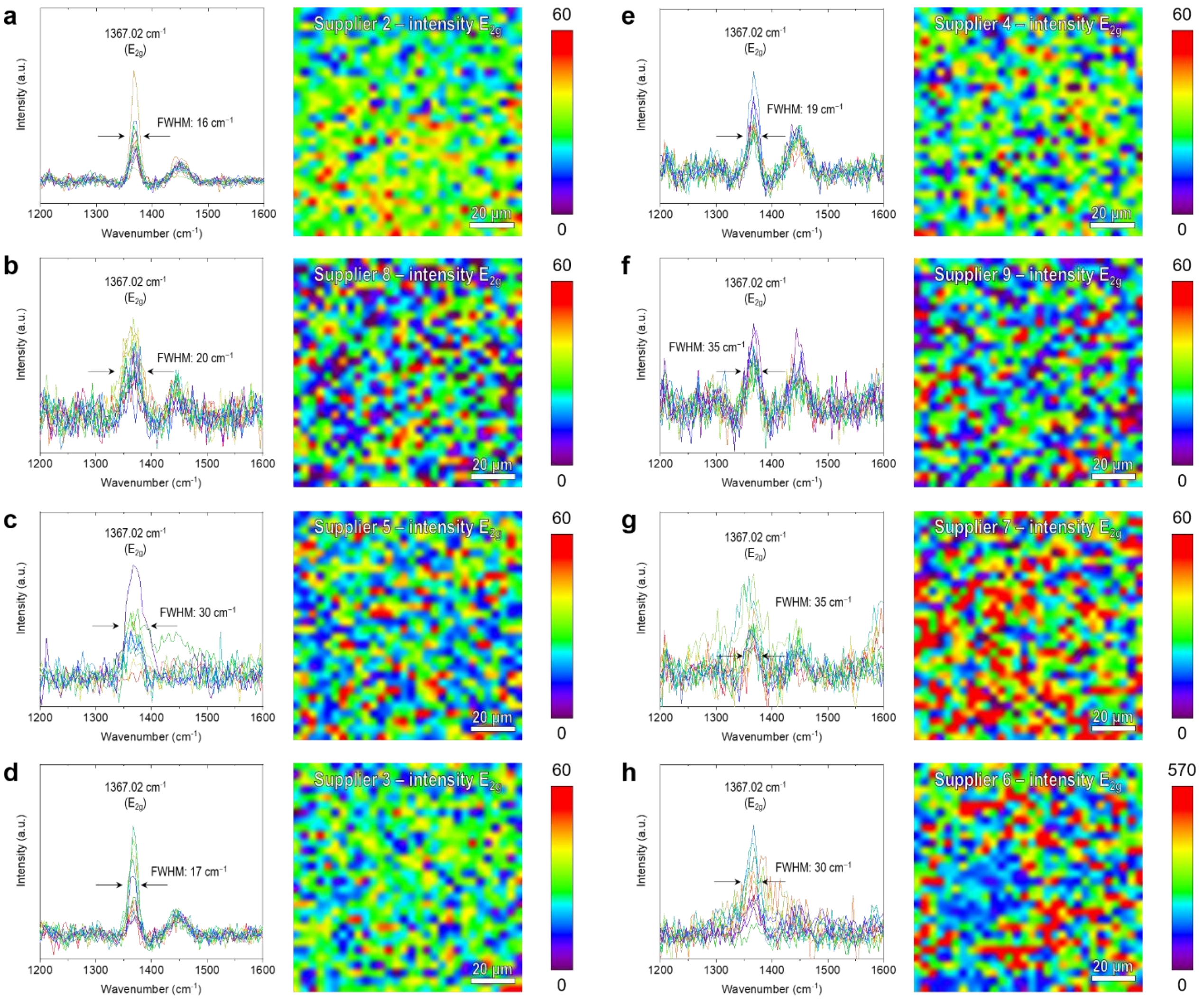

Supplementary Figure 16 | Raman characterization of CVD-grown h-BN samples labeeled as monolayer from Suppliers 2-9, transferred on 300 nm SiO_2 / Si substrates. Each Raman spectrum plot contains 12 Raman spectrums collected at 12 different positions. Each Raman map is in a size of 100 µm × 100 µm, selected of the intensity of h-BN E_{2g} band. The order from **b** to **h** follows the same order as in Supplementary Figure 8.

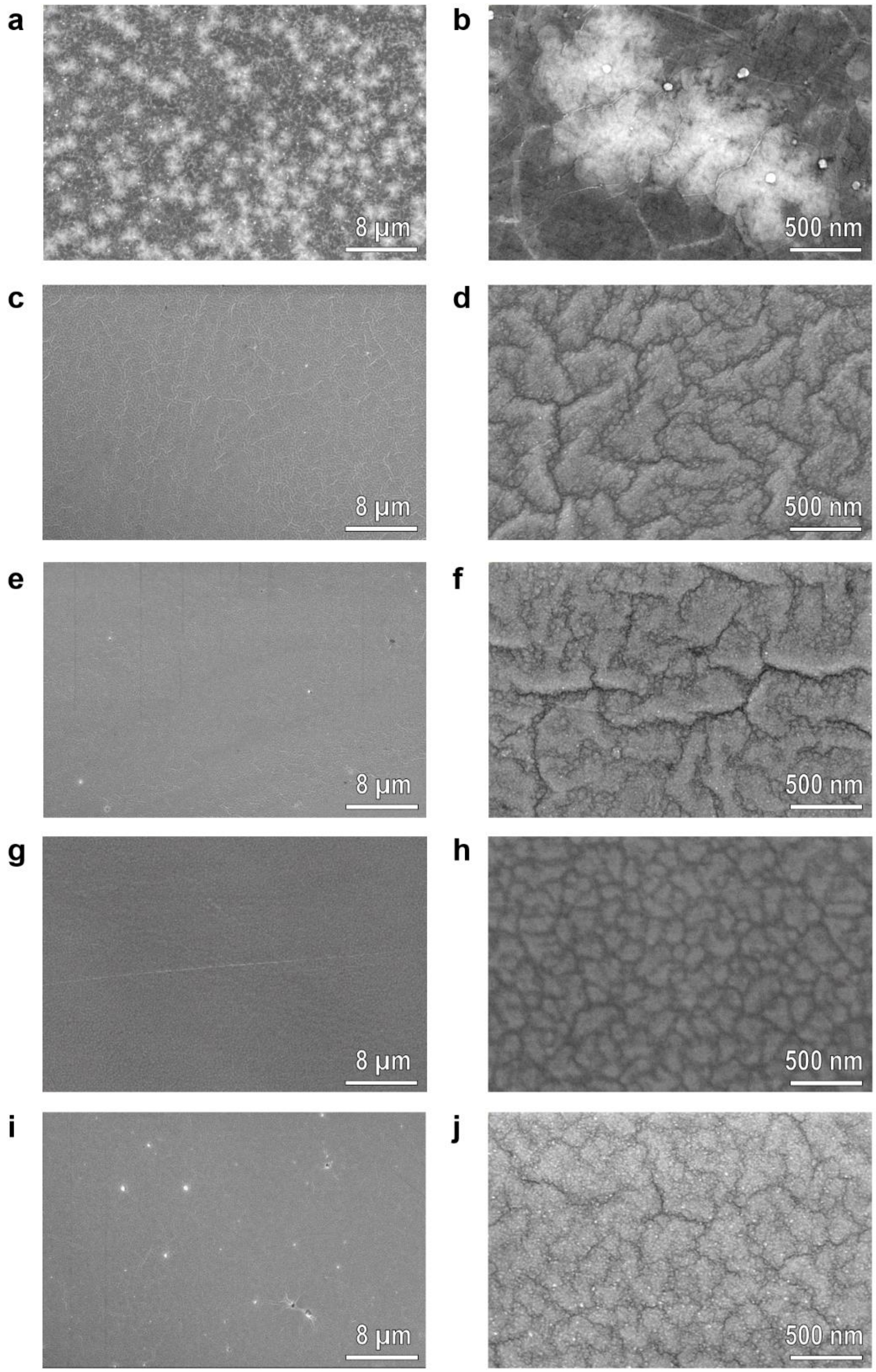

Supplementary Figure 17 | SEM characterization of five CVD-grown multilayer h-BN samples from Supplier 1. We characterize five different samples, each one of them displayed in a different row. The left column shows larger areas, while the right column displays detailed pictures of the corresponding image in the left column (i.e., same row).

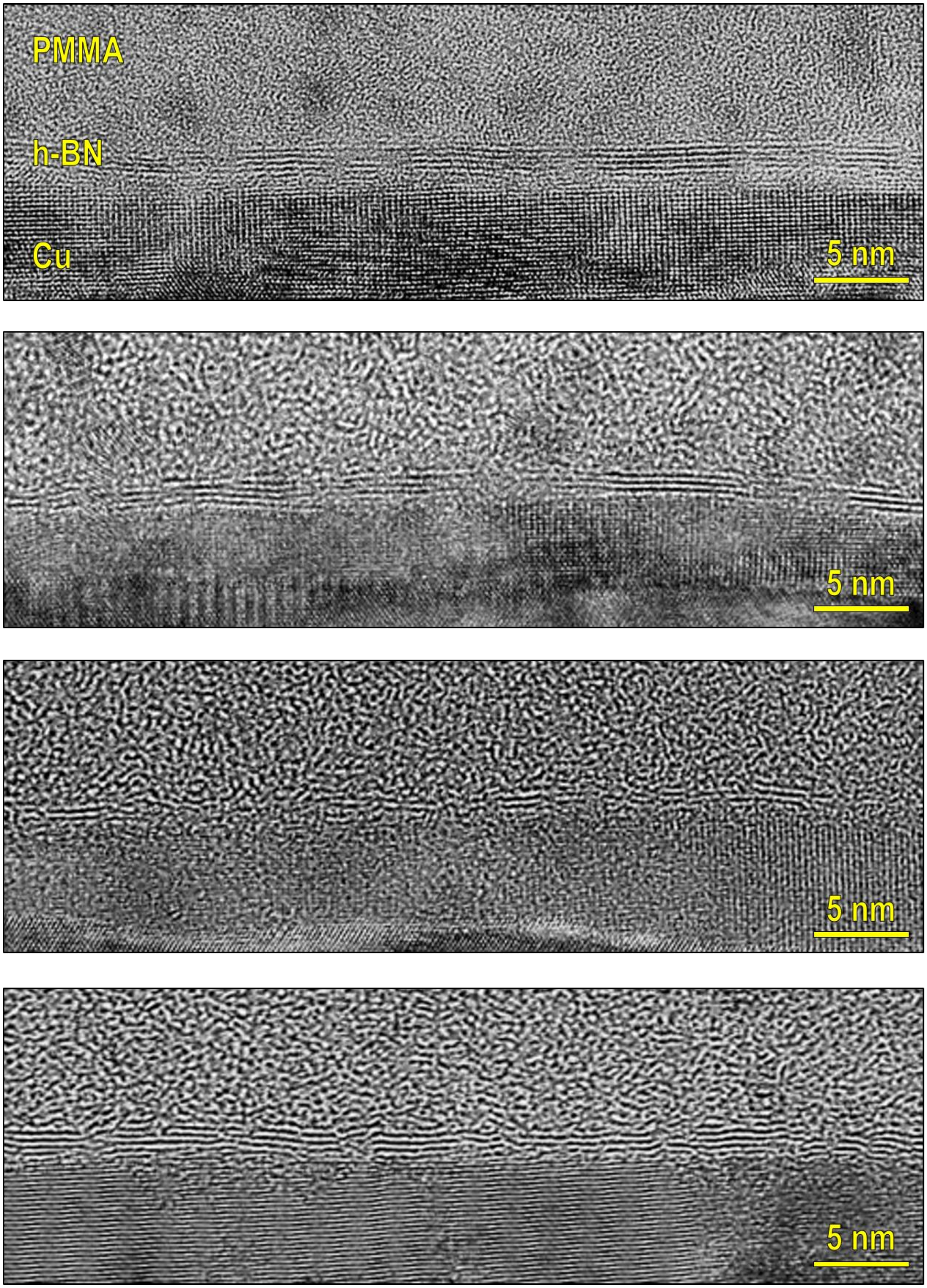

Supplementary Figure 18 | Cross-sectional TEM images of multilayer CVD-grown h-BN on Cu, from Supplier 1 – Sample 1. The PMMA served as protective coating during the FIB cut. The layered structure is obvious, with van der Waals gap between each layer.

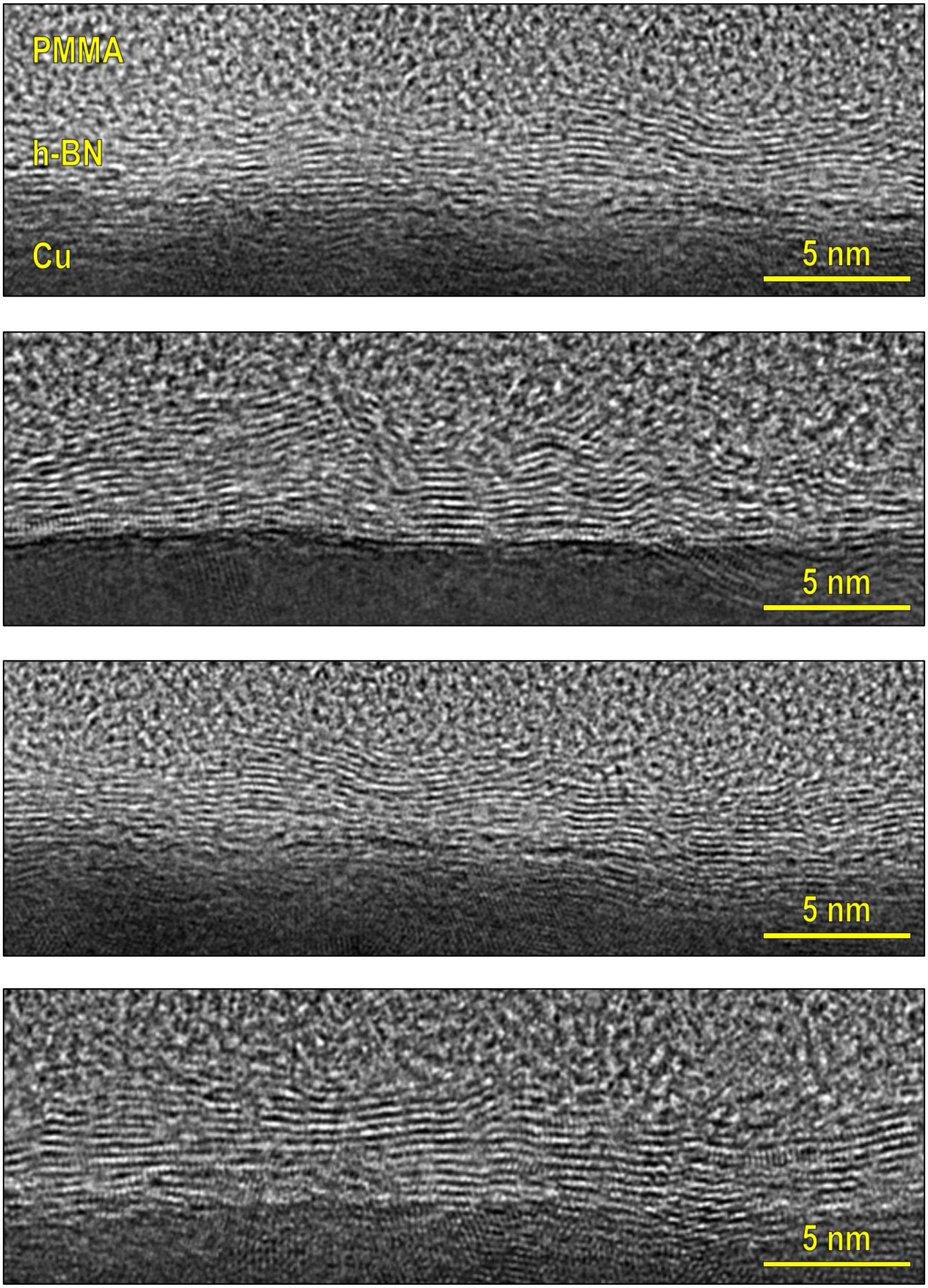

Supplementary Figure 19 | Cross-sectional TEM images of multilayer CVD-grown h-BN on Cu, from Supplier 1 – Sample 2. The PMMA served as protective coating during the FIB cut. The layered structure is obvious, with van der Waals gap between each layer.

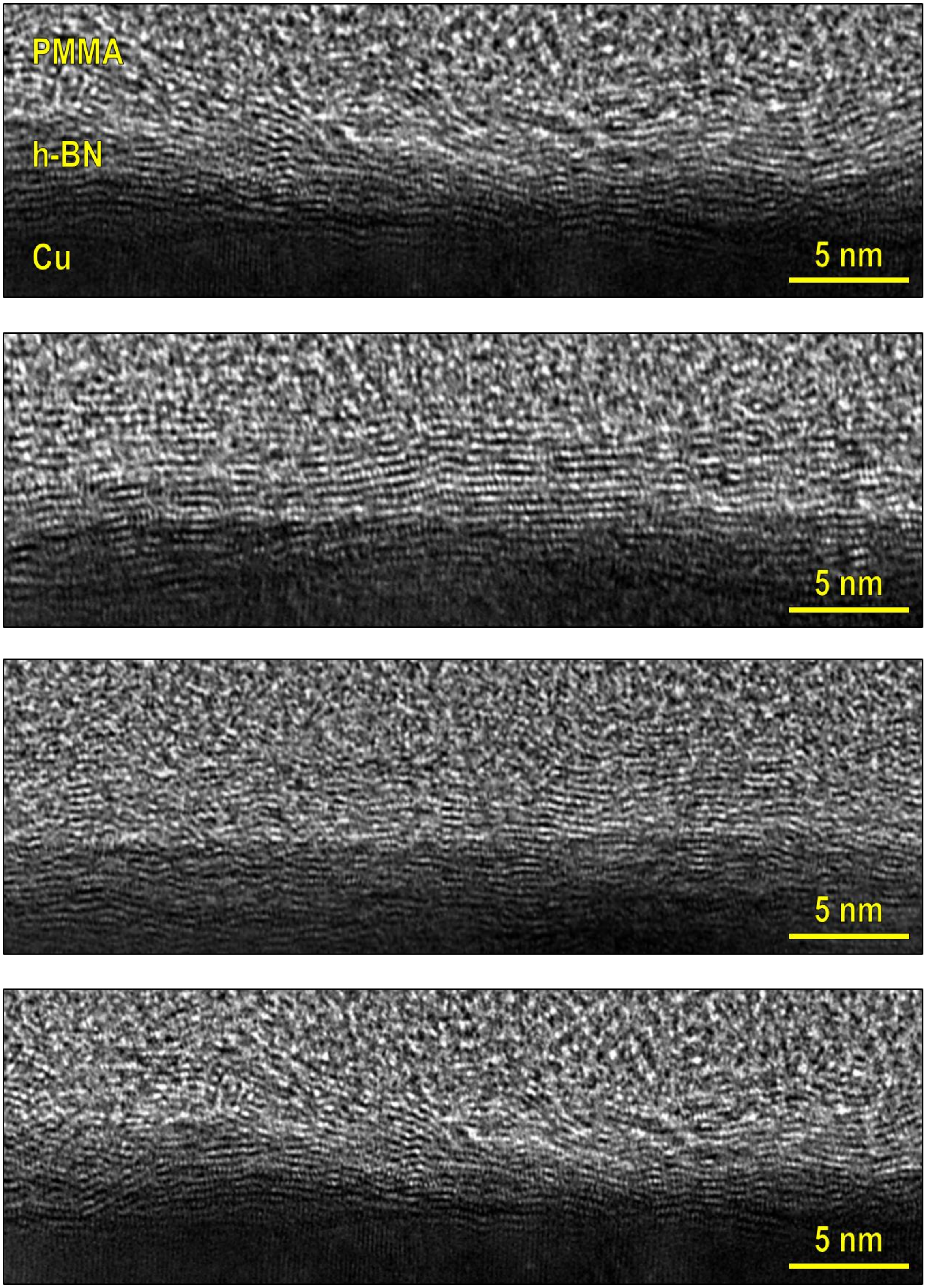

Supplementary Figure 20 | Cross-sectional TEM images of multilayer CVD-grown h-BN on Cu, from Supplier 1 – Sample 3. The PMMA served as protective coating during the FIB cut. The layered structure is obvious, with van der Waals gap between each layer.

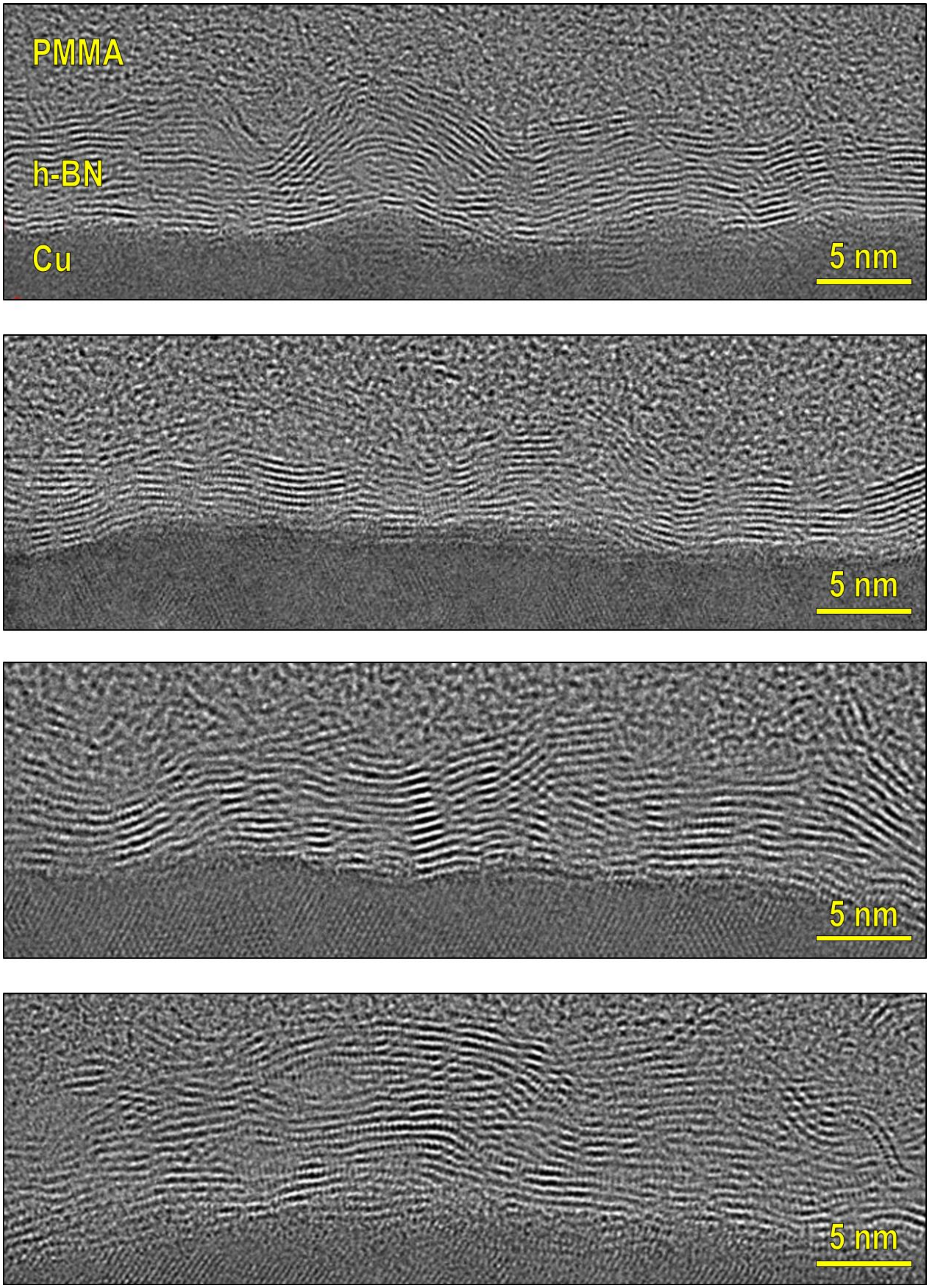

Supplementary Figure 21 | Cross-sectional TEM images of multilayer CVD-grown h-BN on Cu, from Supplier 1 – Sample 4. The PMMA served as protective coating during the FIB cut. The layered structure is obvious, with van der Waals gap between each layer.

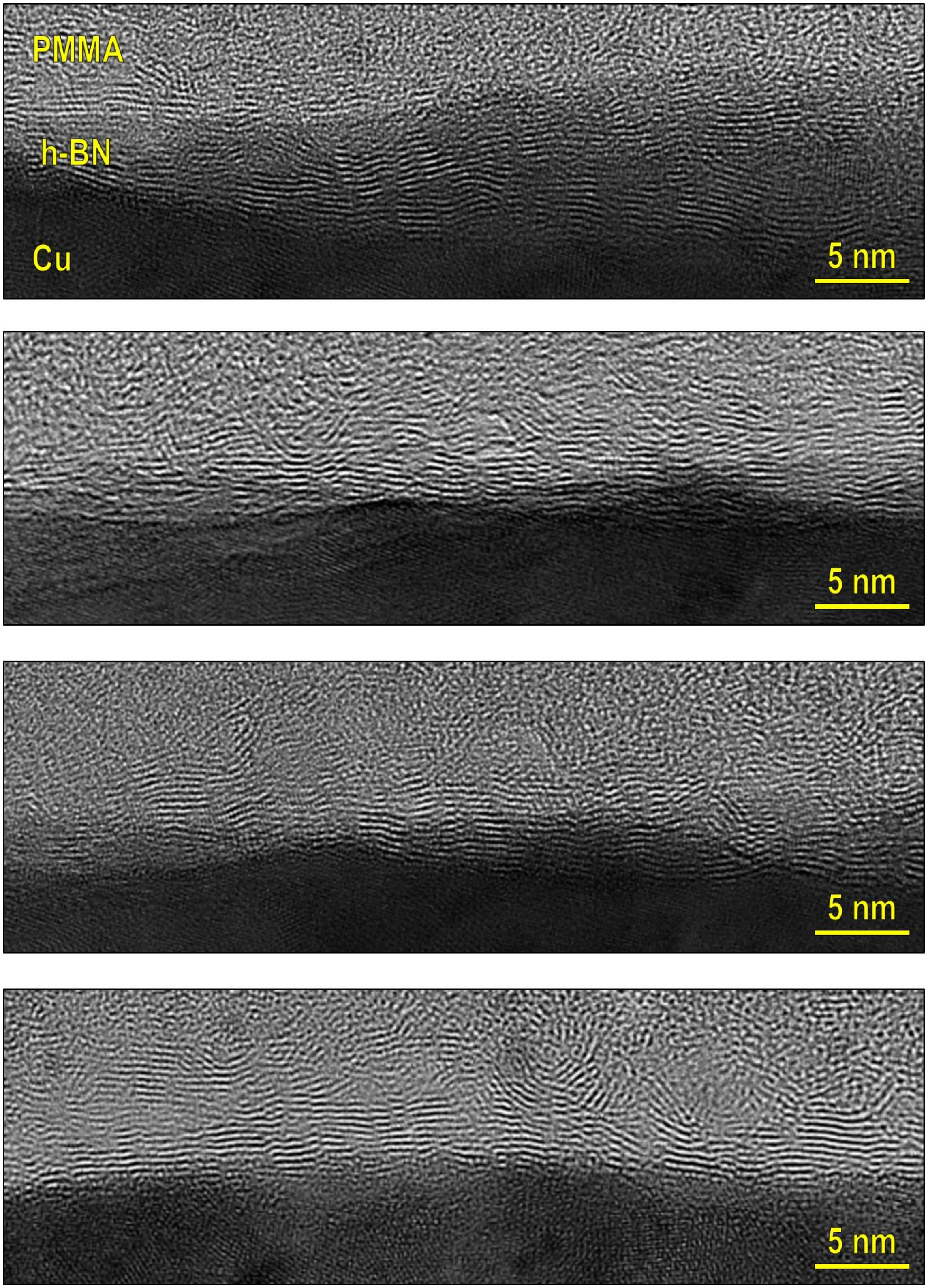

Supplementary Figure 22 | Cross-sectional TEM images of multilayer CVD-grown h-BN on Cu, from Supplier 1 – Sample 5. The PMMA served as protective coating during the FIB cut. The layered structure is obvious, with van der Waals gap between each layer.

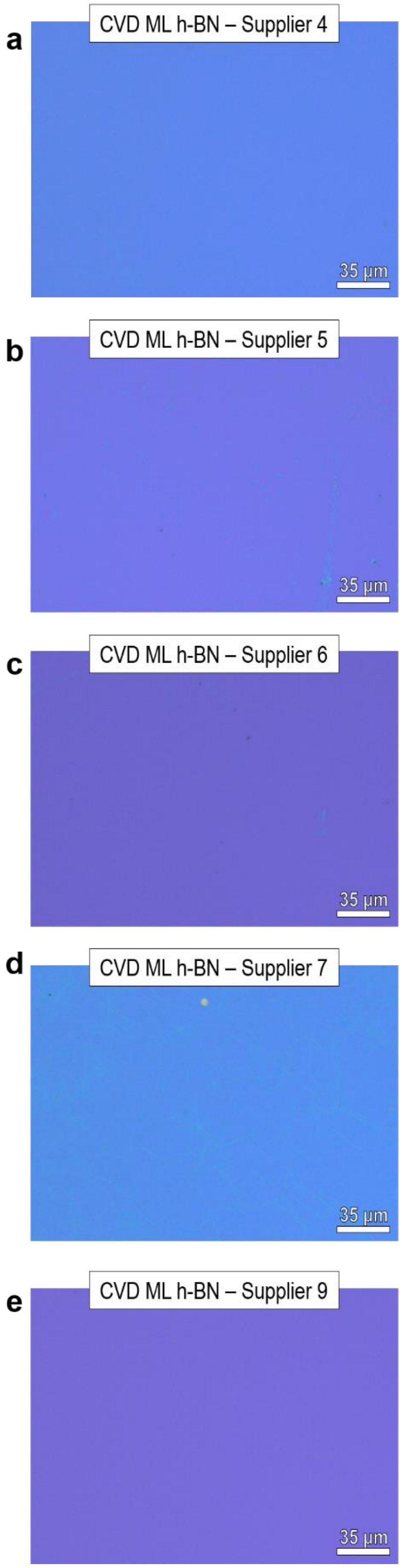

Supplementary Figure 23 | Optical microscope images of CVD-grown multilayer h-BN from Suppliers 4, 5, 6, 7, and 9, transferred on 300 nm SiO_2 / Si substrates.

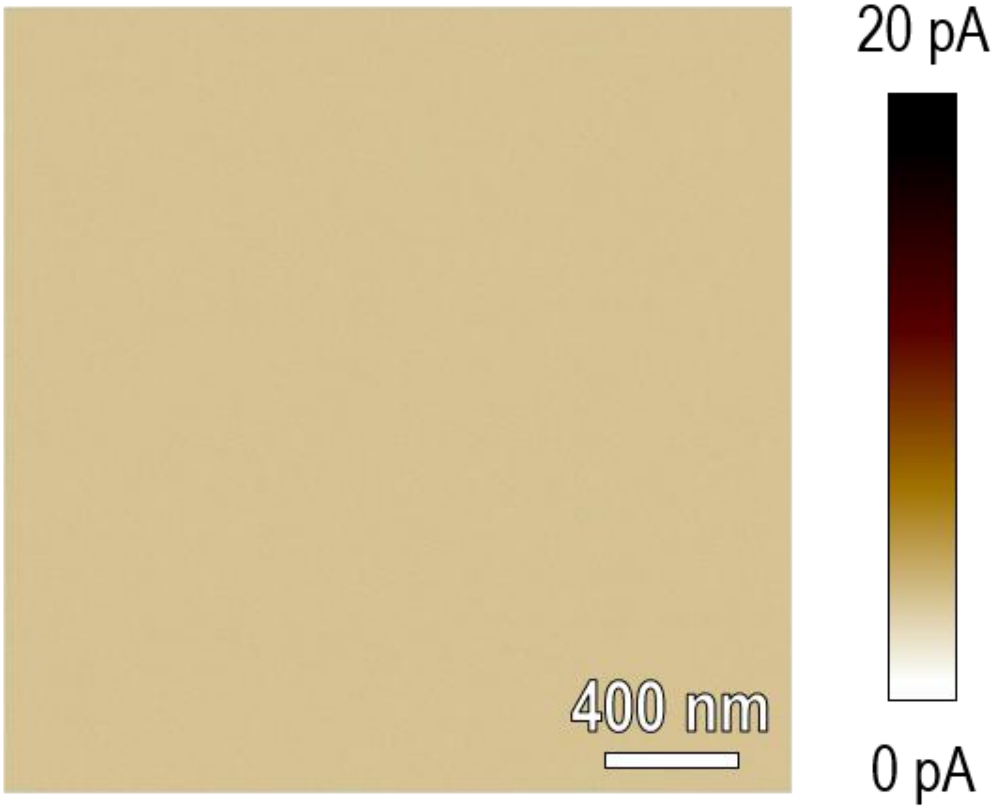

Supplementary Figure 24 | Pinhole analysis of multilayer CVD h-BN. CAFM current map of CVD-grown multilayer h-BN on Cu foil, from Supplier 1. This current map was collected while there was no bias applied between the sample and the tip. No conductive current spot can be observed.

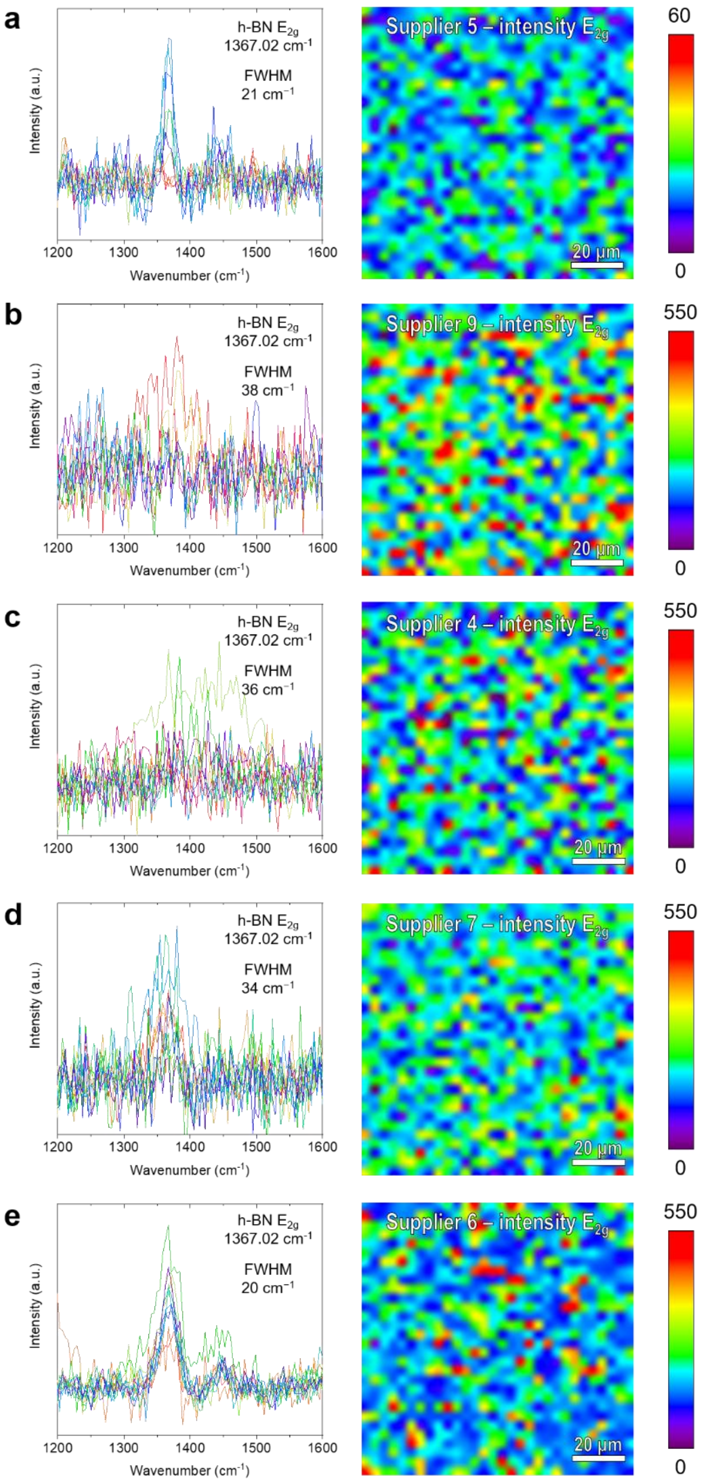

Supplementary Figure 25 | Raman characterization of CVD-grown multilayer h-BN from Supplier 2, 4, 7, 9 and 12, transferred on 300 nm SiO_2 / Si substrates. Each Raman spectrum plot contains 12 Raman spectrums collected at 12 different positions. Each Raman map is in a size of 100 µm × 100 µm, selected of the intensity of h-BN E_{2g} band. The order from **a** to **e** follows the same order as in Figure 5.

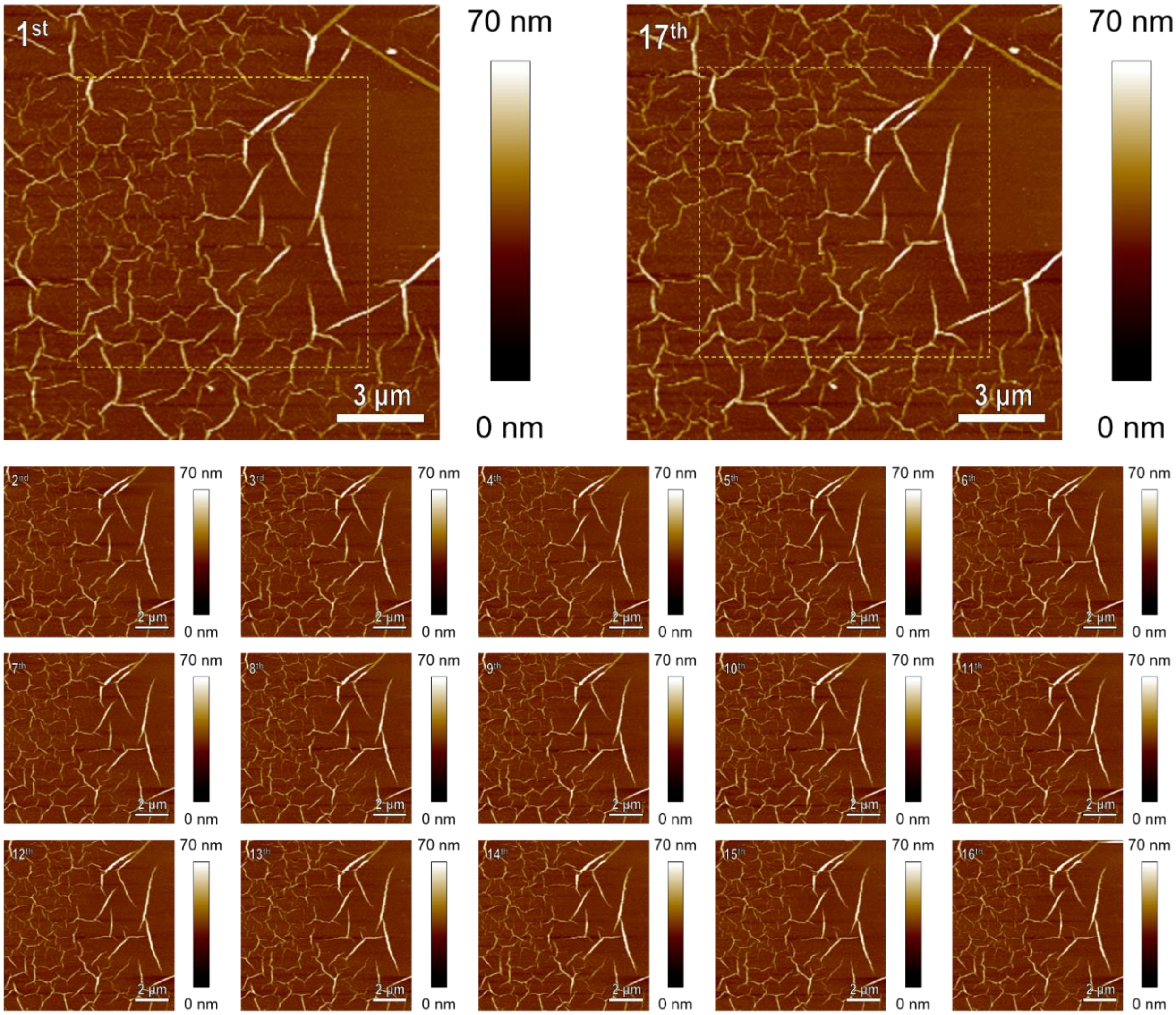

Supplementary Figure 26 | CAFM topography maps collected in sequence on multilayer h-BN samples. The 1st scan and the last scan (17th) are a zoom out topography maps with size of 15 µm × 15 µm, while the 2nd ~ 16th are taken at the centre of the sample.
